# Dysphagia Megalatriensis: An Uncommon Cardiac Mimicker of Gastroesophageal Dysphagia

**DOI:** 10.14309/crj.0000000000001374

**Published:** 2024-06-03

**Authors:** Leonard Palatnic, Ross Robert Moyer, Clive Jude Miranda

**Affiliations:** 1 Department of Gastroenterology, Jacobs School of Medicine and Biomedical Sciences, State University of New York at Buffalo, Buffalo, NY; 2 Department of Cardiology, Jacobs School of Medicine and Biomedical Sciences, State University of New York at Buffalo, Buffalo, NY

**Keywords:** cardiac dysphagia, endoscopy, echocardiogram

## CASE REPORT

**Figure 1.**
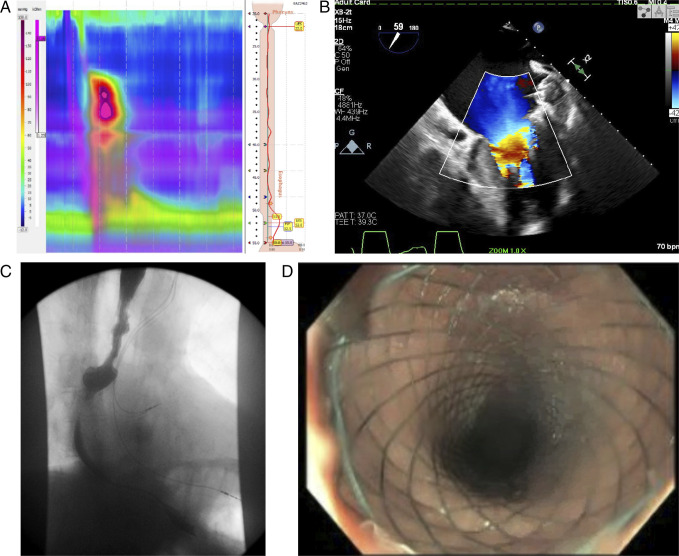
(A) Esophageal manometry showing findings concerning for grade III achalasia. (B) Echocardiogram revealing severe left atrial dilation and bowing of the interatrial septum. (C) Upper gastrointestinal series revealing esophageal dysmotility and pulsion diverticulum in the mid-esophagus just above the left atrium. (D) Post-esophageal stent placement.

An 82-year-old man with permanent atrial fibrillation status after atrioventricular nodal ablation and Watchman device placement presented for recurrent abdominal pain and dysphagia unresponsive to dietary modification or medications. He underwent esophageal manometry testing, which demonstrated findings concerning for grade III achalasia (Figure 1). To rule out pseudoachalasia, a computed tomography angiogram of the torso was obtained, revealing left atrium (LA) enlargement with mass effect causing compression of the lower one-third of the esophagus, resulting in mild obstruction and dilation of the proximal esophagus. A transthoracic echocardiogram then demonstrated severe LA dilation and bowing of the interatrial septum consistent with elevated LA pressures (Figure 1). With continued dysphagia, an upper gastrointestinal series revealed esophageal dysmotility and pulsion diverticulum in the mid-esophagus just above the LA (Figure 1). The patient underwent an esophagogastroduodenoscopy with esophageal stent placement (Figure 1). Notably, extrinsic compression of the mid-esophagus at the level of the LA was noted peri-procedure. This case illustrates the importance of always considering cardiac disease as an underlying etiology for persistent dysphagia. First described in 1969, dysphagia megalatriensis remains a challenging diagnosis.^1^ However, cardiac workup is warranted when there is true clinical suspicion.

## DISCLOSURES

Author contributions: L Palatnic, RR Moyer, and CJ Miranda compiled a literature review and wrote up the manuscript. CJ Miranda is the article guarantor.

Financial disclosure: None to report.

Previous presentation: Case presented at the American College of Gastroenterology Annual Scientific Meeting; October 23, 2023; Vancouver, BC, Canada.

Informed consent was obtained for this case report.
